# Status of insecticide susceptibility in *Anopheles arabiensis *from Mwea rice irrigation scheme, Central Kenya

**DOI:** 10.1186/1475-2875-5-46

**Published:** 2006-06-06

**Authors:** Luna Kamau, John M Vulule

**Affiliations:** 1 Centre for Biotechnology Research and Development, Kenya Medical Research Institute, P.O. Box 54840, Nairobi-00200, Kenya; 2 Centre for Vector Biology and Control Research, Kenya Medical Research Institute, P.O. Box 1578, Kisumu-40100, Kenya

## Abstract

**Background:**

Control of the Anopheline mosquito vectors of malaria by use of insecticides has been shown to impact on both morbidity and mortality due to this disease. Evidence of insecticide resistance in different settings necessitates surveillance studies to allow prompt detection of resistance should it arise and thus enable its management. Possible resistance by *Anopheles arabiensis *mosquitoes from Mwea rice irrigation scheme in Central Kenya to insecticides in the four classes of insecticides approved by WHO for indoor residual spraying was investigated.

**Methods:**

Susceptibility to DDT (an organochlorine), fenitrothion (an organophosphate), bendiocarb (a carbamate), lambdacyhalothrin and permethrin (both pyrethroids) was tested using standard WHO diagnostic bioassay kits. Bioassays were performed on non-blood fed mosquitoes one- to three-day old. Knockdown was recorded every 10 min and mortality 24 h post-exposure was noted.

**Results:**

Mortality 24 h post-exposure was 100% for all insecticides except for lambdacyhalothrin, which averaged 99.46%. Knockdown rates at 10 min intervals were not significantly different between the Mwea population and the susceptible KISUMU strain of *Anopheles gambiae *sensu stricto control. The KDT_50 _and KDT_95 _values for the Mwea population were either lower than those for the control or higher by factors of no more than 2 for most comparisons and compared well with those of *An. gambiae *sensu lato categorized as susceptible in other studies.

**Conclusion:**

These results suggest that the Mwea population of *An. arabiensis *is susceptible to all the insecticides tested. This implies that vector control measures employing any of these insecticides would not be hampered by resistance.

## Background

*Anopheles gambiae *sensu stricto, *Anopheles arabiensis *and *Anopheles funestus *are the most important vectors of malaria in sub-Saharan African and occur in sympatry across most of their range [1]. Studies show that the use of insecticides both for Indoor Residual Spraying (IRS) programmes and in the treatment of bed nets has resulted not only in a reduction in vector population densities but also in morbidity and mortality due to malaria [2-4]. There is, however, evidence that malaria vectors are developing resistance to commonly used insecticides [5]. In Western Kenya, resistance was first reported in the context of Insecticide-Treated Net (ITN) use [6]. Although more recent studies indicate that resistance levels have increased only marginally [7], there is concern that continued and/or increased use of insecticides may result in increased resistance that would threaten the sustainability of this vector control strategy. Insecticide resistance is more widespread in West Africa where it has been associated with use of insecticides in public health for mosquito control and in agriculture for pesticide control [8-11]. Levels of insecticide resistance have been shown to vary even within relatively small geographical scales and during different seasons [9,10]. The dominant resistance mechanisms also vary as was observed in Guatemalan populations of *Anopheles albimanus*, where both insecticide resistance levels and mechanism varied within short distances [12]. These observations suggest the shifting nature of insecticide resistance and imply therefore that extrapolations from one circumstance to another may be misleading. Studies in Haitian populations of *An. albimanus *found resistance frequencies to fenitrothin to increase from 20 to 60% over a period of six months [13] and underscore the need for continuous insecticide resistance monitoring, even where no evidence of resistance has previously been found.

The current study presents the first report on the status of insecticide resistance/susceptibility in a rice-irrigation scheme in Central Kenya. Resistance was tested against insecticides in each of the four classes that have been approved for IRS by WHO. The results of this study will enable informed selection of insecticides for vector control programmes as well as provide baseline information essential in the monitoring of the development of insecticide resistance.

## Materials and methods

### Study area and insecticide use patterns

The study was carried out in Mwea area (00° 67'S, 37° 35'E) of Central Kenya. This is predominantly a rice-growing area although other crops such as beans, maize and green vegetables are grown for subsistence. Previously, rice was grown during a single growing season that extended from June to December but in recent years, different paddies are flooded intermittently during the year due to water shortages associated with the prevailing drought, thus maintaining almost all-year-round rice growing although the main growing season is still from June to December.

A survey to establish insecticide/pesticide-use patterns in the study area was conducted. This was done by administering a simple questionnaire on the pesticides used in agriculture and their concentrations and whether residents used bed nets and if they did, whether the bed nets were insecticide-treated. A total of 42 households were surveyed.

### Specimen collection, identification and rearing

Specimens were collected both as larvae from rice paddies using standard dippers and as adults by aspiration from walls inside human dwellings. Collections were made on 4^th ^and 5^th ^August 2004 and again on 9^th ^and 10^th ^September 2004 during the dry season, which coincided with the main rice growing season and most paddies were flooded, and then again during the rainy season between 3^rd ^– 5^th ^May 2005. Specimens were identified as *An. gambiae s.l*. based on morphological characteristics [1]. Larvae from the different paddies were preserved live in separate bottles and transported to the insectary for rearing. The larvae were then reared into adults as follows: a single larval specimen was picked from each of the transportation bottles and placed in a rearing pan so that each pan contained just one specimen from each rice paddy. This was done to limit the chances that siblings were included in individual bioassay runs and thus obtain better estimates of population variability in insecticide susceptibility. Six pans were constituted in this manner and the resulting adults used for each of the five insecticide bioassays that were run and the sixth for the control test using untreated test paper. For the specimens collected as adults, individual field-collected females were allowed to oviposit and F1 families raised separately. Only one specimen from each of the families was used in each of the bioassays. Specimens were identified further to sibling species of the *An. gambiae *complex using species-specific Polymerase Chain Reaction technique [14] after DNA extraction by the alcohol precipitation method [15]. Field-collected adults were identified after they had oviposited while specimens collected as larvae were identified after the insecticide resistance bioassays were performed.

### Insecticide susceptibility bioassays

Insecticide susceptibility assays were performed on adult non-blood fed mosquitoes one- to three-day old that were reared from field-collected larvae as described above or on F1s of field-collected adult mosquitoes. The tests were carried out using 4% DDT, 1% fenitrothion, 0.1% bendiocarb, 0.05% lambdacyhalothrin and 0.75% permethrin, the diagnostic doses recommended by WHO. The Bioassay kit, Mosquito (Adult) Diagnostic test kit WHO/VBC/81.806, was supplied by Universiti Sains Malaysia (USM), Penang, Malaysia and the assay carried out according to the accompanying instructions. Briefly, for each of the insecticides tested, mosquitoes were divided into batches between 15–25 mosquitoes and exposed to insecticide-treated papers for 1 h for DDT, bendiocarb and permethrin and for 2 h fenitrothion and lamdacyhalothrin. Insecticide knockdown effects were recorded every 10 min until 100% knockdown was observed. At the end of the exposure period, mosquitoes were transferred into tubes with untreated papers and allowed a 24 h recovery period after which mortality was recorded. Tests were accompanied by control tests where mosquitoes were exposed to papers treated only with silicone oil for 1 h or 2 h depending on the insecticide that was being tested against. Bioassays were also carried out on the *An. gambiae *KISUMU susceptible strain (KSM Strain). Mortality was noted 24 h post exposure as defined in the criteria for determining resistance or susceptibility to diagnostic doses of insecticide. All mosquitoes were supplied with a 6% glucose meal during the 24 h recovery period.

### Statistical analyses

Mean mortality was determined across all batches of mosquitoes tested for a particular insecticide and the WHO [5] criteria used to evaluate the resistance/susceptibility status of the mosquito tested. By the said criteria, resistance is indicated by mortality rates of less than 80% 24 h after exposure to insecticide while mortality rates greater than 98% are indicative of susceptibility.

Mortality rates between 80–90% suggest the possibility of resistance that needs to be clarified. Knockdown rates at 10 min intervals for the Mwea larval and adult collections for each of the insecticides tested and for the dry and rainy season collections were compared using the paired *t-*test. Knockdown rates at 10 min intervals were also compared between the Mwea mosquito collections and the KSM strain using the paired *t*-test. Fifty and 95% knockdown times (KDT_50 _and KDT_95 _respectively) for both the Mwea collection and the KSM strain were estimated by the log-time probit model using the *LdP Line*^R ^software [16]. The fit of the probit model was assessed using chi-square distribution analysis and the Bonferroni Procedure used to determine the overall significance of multiple tests.

## Results

All households interviewed said that they had used fenitrothion as a pesticide in rice growing for at least the last ten years but did not know the concentration at which it was used. An interview with a manager at the Mwea Rice Growers Multipurpose Co-operative Society, the organization that supplies the pesticides to the farmers and through which the farmers sell their produce revealed that fenitrothion alongside carbofuran have been the pesticides in use for agricultural spraying but the use carbofuran was stopped two years prior to the study due to cost factors. Fenitrothion 50 EC is used at a concetration of 0.5% and is sprayed onto two-week old rice seedlings in the nursery and again 21–28 days after transplanting. The survey also revealed that no organized vector control programmes are available in the study area but that approximately 93% of the 42 households surveyed used bed nets. Of the total number of bed nets used, 39% were pyrethroid (deltamethrin)-treated but bed nets were not retreated after purchase. Approximately 55% of household also use either pyrethroid aerosol sprays or mosquito coils. The use of the aerosols and mosquito coils is higher during the rainy and the rice-growing season when the residents perceive that mosquito densities are high and thus also the threat of malaria.

The total numbers of field-collected specimens that were tested for each of the five insecticides are shown in the table. In addition, a total of 821 mosquitoes belonging to 49 families (family size 10–73 mosquitoes) were tested for susceptibility to lambdacyhalothrin after initial results indicated recovery after the 24 h period. All specimens tested were *An. arabiensis *by the specific-specific PCR assay.

Mortality, after the 24 h recovery period, was 100% for DDT, fenitrothion, bendiocarb and permethrin and for lambdacyhalothrin for the adult collection. Mortality was however slightly reduced for the lambdacyhalothrin assay with the larval collection and with the single-family samples, mortality being 99.1% ± 0.63 S.E.(for a total of 221 mosquitoes tested in 11 batches) and 99.3% ± 0.36 S.E. (for a total of 821 mosquitoes tested in 34 batches) respectively. Mortality on the KSM strain control was 100% for all the insecticides tested except for DDT (see Table 1).

**Table 1:** Percentage mortality and (in brackets) total number of mosquitoes test and KDT_50 _and KDT_95 _values for the different Insecticides tested

Insecticide	Sample	% mortality(n)	KDT_50 _(95% CI)	KDT_95 _(95% CI)	χ^2^
DDT	Mwea	100 (411)	25.51(23.95–27.0)	49.89(46.44–54.32)	3.73^ns^
(4%)	KSM Strain	99.23 (130)	62.51(56.94–70.45)	210.56(161.84–309.73)	6.84^ns^
					
Fenitrothion	Mwea	100 (405)	55.02(46.81–62.39)	95.1(88.89–121.83)	91.28*
(1%)	KSM Strain	100 (131)	88.90(83.68–95.20)	130.40(123.56–151.10	25.38*
					
Bendiocarb	Mwea	100 (366)	21.31(19.95–22.61)	37.85(35.06–41.65)	2.4^ns^
(0.1%)	KSM Strain	100 (127)	14.10(13.19–15.05)	22.87(20.93–25.66)	0.23^ns^
					
Lambda-C^a^	Mwea	99.61(525)	21.58(20.09–23.01)	43.00(39.53–47.57)	12.55^ns^
(0.05%)	KSM Strain	100 (119)	19.78(15.61–23.39)	57.42(48.96–78.73)	19.78*
					
Permethrin	Mwea	100 (429)	17.24(15.94–18.49)	34.77(31.75–38.90)	2.01^ns^
(0.75%)	KSM Strain	100 (123)	27.75(22.31–32.88)	66.96(58.66–98.11)	14.5*

^a^Lambdacyhalothrin

χ2 values are for the test of fit of the log-time probit model used to estimate the KDT_50 _and KDT_95_values; ns = deviations not significant; * = deviations significant, P < 0.05

**Figure 1:**
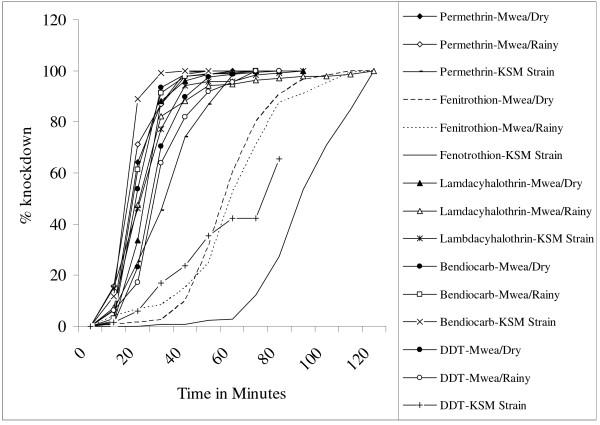
**Percentage knockdown against time for the Mwea *An.arabiensis *population and the *An. gambiae *KISUMU strain**. The figure shows the results of insecticide resistance bioassays using diagnostic doses of each of the insecticides. Results are for mean knockdown across all batches of mosquitoes that were tested for each of the different seasons.

Percentage knockdown at 10 min intervals was not significantly different between the Mwea larval and adult collections for all insecticides tested except for bendiocarb (paired t = 2.9896, df = 4, P = 0.0404). Data for the larval and adult assays were therefore merged for all comparisons except for this insecticide for all subsequent analyses. Percentage knockdown rates at 10 min intervals were also not significantly different between the dry and the rainy season collections nor between the Mwea collections and the KSM Strain for each of the insecticides tested (paired t, P > 0.05 in all cases). Figure 1 shows percentage knockdown versus exposure time for the Mwea collections and for the KSM Strain for each of the insecticides tested. The log-time probit model used to estimate KDT_50 _and KDT_95 _values did not fit the distribution of percentage knockdown with time for the fenitrothion assay for the Mwea collections or for the KSM Strain for the fenitrothion, lambdacyhalothrin and permethrin bioassays (P values for the chi-square test of heterogeneity <0.05 in each of these cases; P values were also significant for the global test). The KDT_50 _and KDT_95 _estimates in these cases were not therefore included in the comparisons as these would be unreliable although they are given in the table. All other KDT_50 _and KDT_95 _values for the Mwea population were either lower than those for the KSM Strain or only increased slightly, by factors of less than two.

## Discussion

Overall, the results obtained in this study suggest good susceptibility of *An. arabiensis *in the study area to all the five insecticides tested. This means that vector control programmes employing any of these compounds either in the treatment of bed nets or other materials or for indoor residual spraying would achieve satisfactory success rates. This is especially important as *An. arabiensis *was the only member of the *An. gambiae *complex found in the study area, a finding consistent with earlier studies in the area by researchers who found this species to constitute 87.3% of all Anopheline mosquitoes collected [17].

Based on the WHO criteria for characterizing insecticide resistance/susceptibility, where susceptibility is defined by mortality rates greater than 98% 24 h post-exposure, no evidence for resistance to any of the insecticides tested was found. Knockdown rates at 10 mins intervals were not significantly different between the Mwea collections and the KSM susceptible strain. In addition, KDT_50 _and KDT_95 _observed in the present study compare well with those from other studies for *An. gambiae s.l*. populations that are categorized as susceptible [9,18,19]. It is interesting to note that despite the high level of compliance and long-term use of fenitrothion, the *An. arabiensis *mosquito population has not developed resistance to this chemical. A possible explanation is that its levels in agricultural use are below what would select for possible naturally occurring resistance in this species.

The zero or near-zero levels of insecticide resistance in *An. arabiensis *that were observed in the present study are similar to those recently reported from an area of long-term ITN use in Western Kenya based on the presence of the knockdown resistance (*kdr*) gene [7]. The *kdr *mechanism results from mutations in the voltage-gated sodium channel, the target-site for DDT and pyrethroids and is one of the two most important forms of biochemical resistance mechanisms, the other being metabolic resistance, which occurs when levels of insecticide-detoxifying enzymes are elevated or their activity modified [20]. Similarly low or no resistance to pyrethroid insecticides and DDT caused by the *kdr *mutation has been observed within the M form of *An. gambiae s.s*. and *An. arabiensis *in several West African countries despite significant levels of resistance being found within the S form of *An. gambiae s.s*. [8,9,18-21]. The situation was however found to be different in South Africa where significant levels of resistance to DDT in *An. arabiensis*, by the WHO [5] criteria, were observed [22]. Earlier studies in the Sudan also found significantly high resistance levels to malathion in *An. arabiensis *[23], suggesting that this species is not immune to the development of resistance. These differences re-emphasize the focal nature of insecticide resistance and the need to carry out situation analyses and monitoring for individual settings. In Western Kenya, for example, the frequency of the *kdr *gene was found to increase albeit marginally four years after the introduction of ITNs but remained unchanged in villages 20 km away [7]. Studies to assess the effect of longer-term use of the ITNs on resistance in this area are crucial. In Burkina Faso, resistance levels were found to vary not only between villages within 100 km of each other and between different seasons but also to different insecticides, with resistance being seen to DDT but not to permethrin [9]. The Western Kenya and most of the West African studies, however, assayed only for the presence of the *kdr *gene to the exclusion of other possible resistance mechanisms. It would be interesting to obtain data on the levels of phenotypic resistance comparison. Brogdon and McAllister [20] have however argued that for insecticide resistance to be a concern, the level of resistance must be high enough to compromise the efficacy of intervention programmes employing the insecticides for vector control. It is controversial though what such a level would be given that studies in Côte d'Ivoir, for example, found nets impregnated with permethrin or deltamethrin to provide good levels of protection where the frequency of the kdr allele was 94% kdr [24].

## Conclusion

These findings suggest that the *An. arabiensis *populations from Mwea are susceptible to all the insecticides that were tested against and therefore that vector control effort utilizing any of these insecticides would not be compromised by resistance. Thus, the results obtained in this study will enable informed choice of insecticides for use in vector control programmes in the area. In addition, the data obtained will provide baseline information needed in the monitoring of the development of resistance to the insecticides arising either due to selective pressure from the use of insecticides and pesticides or through migration to the area of mosquitoes with insecticide resistance genes.

## Authors' contributions

LK conceived and designed the study, carried out the insecticide resistance bioassays, data analysis and interpretation and prepared the manuscript. JV participated in the development of the study design, carried out interpretation of the data and provided a critical review of the manuscript.
